# Alcohol and the Brain

**DOI:** 10.3390/nu13113938

**Published:** 2021-11-04

**Authors:** David Nutt, Alexandra Hayes, Leon Fonville, Rayyan Zafar, Emily O.C. Palmer, Louise Paterson, Anne Lingford-Hughes

**Affiliations:** Neuropsychopharmacology Unit, Division of Psychiatry, Department of Brain Sciences, Hammersmith Hospital, Imperial College London, London W12 ONN, UK; alexandra.hayes17@imperial.ac.uk (A.H.); l.fonville@imperial.ac.uk (L.F.); r.zafar19@imperial.ac.uk (R.Z.); e.palmer@imperial.ac.uk (E.O.C.P.); l.paterson@imperial.ac.uk (L.P.); anne.lingford-hughes@imperial.ac.uk (A.L.-H.)

**Keywords:** alcohol, brain, addiction, alcoholism

## Abstract

Alcohol works on the brain to produce its desired effects, e.g., sociability and intoxication, and hence the brain is an important organ for exploring subsequent harms. These come in many different forms such as the consequences of damage during intoxication, e.g., from falls and fights, damage from withdrawal, damage from the toxicity of alcohol and its metabolites and altered brain structure and function with implications for behavioral processes such as craving and addiction. On top of that are peripheral factors that compound brain damage such as poor diet, vitamin deficiencies leading to Wernicke-Korsakoff syndrome. Prenatal alcohol exposure can also have a profound impact on brain development and lead to irremediable changes of fetal alcohol syndrome. This chapter briefly reviews aspects of these with a particular focus on recent brain imaging results. Cardiovascular effects of alcohol that lead to brain pathology are not covered as they are dealt with elsewhere in the volume.

Terminology describing alcohol consumption has varied over the years but generally drinking at a level causing mental and/or physical ill health is referred to as harmful (ICD-10/11) or abuse (DSM-IV). Alcohol dependence or addiction or alcoholism is a complex behavioral syndrome that has at its core the inability to control consumption despite adverse social, occupational or health consequences (ICD-10/11; DSM-IV). More recently DSM-5 criteria use the term alcohol use disorder (AUD) as a continuum with mild equivalent to abuse and moderate-severe equivalent to dependence [1]. Whilst most social drinkers remain in control of their engagement with alcohol, a small but significant minority transition to dependence. Alcoholism is a global public health emergency with the World Health Organization estimating prevalence of 4% and is associated with c. 3 million deaths annually [2]. There is a high rate of relapse; around 80% of dependent individuals relapse within a year with current available therapies [3]. Current adjunctive pharmacotherapies have only mild-moderate effects on alcohol consumption and relapse prevention [4,5] and no there are no rescue medications available to counteract the adverse effects of intoxication [6]. Neuroimaging studies have revolutionized our understanding of brain neurochemistry and function in response to chronic alcohol consumption and dependence, but so far, this has not led to huge improvements in treatment availability or efficacy. In order to improve treatment outcomes, a detailed understanding of the neurobiological mechanisms responsible for vulnerability, relapse and successful recovery and the identification of novel biomarkers to develop more efficacious therapeutic targets are warranted.

## 1. Structural and Volumetric Changes

### 1.1. Structural Alterations in Adults

The link between alcohol use and cerebral atrophy goes back decades, with early findings coming from post-mortem investigations [7] and subsequent in vivo examinations of gross morphology using computerized tomography (CT) [8,9,10]. The emergence of magnetic resonance imaging (MRI) brought vast improvements to image resolution and allowed for differentiation of brain tissue. Whilst the early CT scans pointed towards ventricular enlargement and widening of cortical sulci, MRI studies have provided a wealth of evidence towards distinct differences in grey and white matter associated with chronic alcohol use [11]. Grey matter volume loss is commonly observed in alcohol dependence and effects are widespread across cortical and subcortical regions [12,13], though meta-analytic efforts have pointed specifically towards volume loss in the prefrontal cortex, cingulate cortex, insula, and striatum in particular [14,15,16]. A meta-regression analysis further showed that the impact on grey matter was linked to lifetime alcohol consumption and duration of alcohol dependence [16]. Macrostructural differences can also be observed in white matter volume [17] but more importantly there are noticeable differences in microstructure [18], most notably in the corpus callosum, highlighting potential disruption to myelination and axonal integrity. It should be noted that the impact of alcohol on the cerebellar structure has been relatively understudied and most MRI research has focused on cortical and subcortical structures. The cerebellum is known to be affected in alcohol dependence, even more so in those with additional neurological complications such as Wernicke-Korsakoff syndrome, and studies have found a loss of cerebellar volume that further increases with age [19].

Reductions in brain volume are not necessarily irreversible and early CT studies had already shown that brain volume appears to partially recover with abstinence from alcohol [20,21]. Longitudinal MRI studies further showed that changes to volume follow a non-linear pattern with greater increases occurring in the early stages of abstinence [22,23,24]. Furthermore, reducing alcohol consumption to an average of 20 drinks per month rather than abstaining completely was sufficient to produce increases in brain volume compared with those who returned to patterns of heavy drinking that matched pre-treatment levels (consuming an average of 155 drinks per month) [25]. Though evidence in white matter is limited, it does suggest a similar pattern of recovery with abstinence exists [26,27]. An interesting finding from longitudinal MRI studies has been that people prone to future relapses are distinguishable from those able to abstain [28,29,30,31], suggesting there might be biological differences that play a role in treatment progression.

There is a longstanding notion that alcohol has an interactive effect on the biological aging processes, whereby the brains of alcohol dependent individuals resemble those of chronologically older individuals who do not have alcohol dependence [32]. Imaging studies have long found that the loss of grey matter volume as well as the disturbances to white matter microstructure typically seen in alcohol dependence are exacerbated with age [10,27,33,34,35,36,37,38]. This phenomenon has also been investigated using the brain age paradigm, an approach that investigates healthy brain aging by estimating chronological age from neuroimaging data and examines the difference between an individual’s predicted and actual age [39]. One study found that individuals with alcohol dependence showed a difference of up to 11.7 years between their chronological and predicted biological age based on their grey matter volume [33]. Crucially, the difference showed a linear increase with age and was at its greatest in old age which further offers support to the notion of a greater vulnerability to the effects of alcohol in later life.

Low levels of alcohol consumption have historically been viewed as harmless or even beneficial due to its potentially favorable effects on cardiovascular health [40], as described in more detail elsewhere in this special issue. It has been suggested a similar J- or U-shaped relationship exists with brain structure, but only a few MRI studies have found support for this assumption [41,42,43]. More recent large-scale studies of the general population have in fact shown negative linear associations between alcohol consumption and brain structure, showing widespread reductions in grey and white matter volume as well as white matter microstructure and cortical thickness [44,45,46,47]. Taken together, these studies highlight that there is no protective effect of light drinking (e.g., 1–<7 drinks per week) over abstinence and in fact show, similar to alcohol dependence though to a lesser degree, that any level of alcohol consumption can affect brain volume and white matter microstructure. It should be noted that these are cross-sectional association studies, and it is not possible to infer causality or estimate the impact of alcohol on the brain over time. Furthermore, only a small proportion of the variance in brain structure is explained by alcohol consumption [44,46]. A study on brain age further found that only those who drank daily or almost daily showed a difference between chronological and predicted brain age, and there were no differences in those who drank less frequently or abstained from alcohol [48]. This suggests that whilst low levels of alcohol consumption may affect brain structure, this may not negatively impact healthy brain aging. An important caveat to the generalizability is the relatively small amount of large-scale imaging datasets and the findings discussed here primarily relate to the UK BioBank dataset [44,46,47,48].

### 1.2. Structural Alterations in Adolescents

Alcohol use is typically initiated during adolescence, and studies have found that alcohol can impact neurodevelopmental trajectories during this period. Typical brain maturation can be characterized as a loss in grey matter density due to synaptic pruning alongside ongoing growth of white matter volume that reflects increased myelination to strengthen surviving connections [49]. In adolescents who engage in heavy and binge drinking, as defined by the National Institute on Alcohol Abuse and Alcoholism [50], there is a greater decrease in grey matter volume along with an attenuated increase of white matter volume as well as disturbances in white matter microstructure in comparison to non-drinkers [51]. These effects are found in prefrontal, cingulate, and temporal regions as well as the corpus callosum and may reflect an acceleration of typical age-related developmental processes similar to what we have described in adults with alcohol dependence. Less is known about the dose-response mechanism, though it has been suggested moderate drinking lies somewhere intermediate [52,53]. This would again imply that the impact of alcohol consumption on brain structure is not limited to heavy alcohol consumption. However, it has been noted there are differences in brain structure that predate alcohol initiation and may predispose individuals to heavy alcohol use. Structural precursors have mostly been found in the prefrontal cortex and fronto-limbic white matter and show considerable overlap with structural differences found in individuals with a family history of alcohol dependence [54]. Nevertheless, there are studies that have suggested differences are not solely attributable to familial risk [55,56], and more research is needed to better understand these risk factors.

### 1.3. Pre-Natal Alcohol Exposure

A far more severe disruption to neurodevelopment comes from prenatal exposure to alcohol—collectively known as fetal alcohol spectrum disorders (FASD)—which can encompass cognitive deficiencies, neurobehavioral disorders, growth retardation, craniofacial dysmorphism, and deficits to the central nervous system including brain malformations [57]. Early case studies highlighted striking morphological anomalies, most notably thinning of the corpus callosum and enlargement of ventricles, but subsequent radiological investigations have highlighted there is considerable variability in the impact of FASD on brain development [58]. Quantitative analyses of brain macrostructure in FASD have repeatedly found lower grey and white matter volume along with increased thickness and density of cortical grey matter [59]. Crucially, findings have found no morphological differences in the occipital lobe, suggesting that not all brain structures are affected equally. Brain phenotypes of FASD have consistently been recapitulated in animal models and highlight the modulating role of timing and alcohol exposure [60]. Taken together, it is clear that the teratogenic effects of alcohol on brain structure are widespread and can be seen across the spectrum of FASD. However, understanding the link between these structural alterations and other parameters of FASD remains an ongoing challenge.

### 1.4. Conclusion

In summary, MRI studies have offered invaluable insight into the effects of alcohol and have typically found a loss of volume and reduced myelination throughout the brain. The findings described here fit the notion that alcohol affects healthy brain aging and this effect becomes more pronounced with higher levels of consumption. It also suggests that there may be a greater vulnerability to the effects of alcohol on brain health with old age. The impact of alcohol can be observed early on, moderate to heavy drinking during adolescence leads to observable differences to non-drinkers, but this is further confounded by risk factors to unhealthy drinking patterns and alcohol dependence. However, though MRI research will be important in advancing our understanding of the impact of alcohol on the brain we cannot infer harm solely from alterations to brain structure.

## 2. Neurotoxic Properties of Alcohol

### 2.1. Thiamine Deficiency

Thiamine, also known as Vitamin B_1,_ is an essential cofactor that is critical for nerve function. It is required for the functioning of several enzymes responsible for carbohydrate and lipid metabolism and the generation of several essential molecules including nucleic acids and neurotransmitters [61,62]. The human body is incapable of producing thiamine itself and therefore it must be derived solely from diet. Thiamine deficiency predominantly occurs as a result of malnutrition, and in most western countries is most commonly (90%) associated with alcoholism [63]. Over time, thiamine deficiency can result in nerve damage, leading to alcoholic neuropathy.

Much of the calorific intake of individuals with alcoholism comes from their consumption of alcoholic beverages, which are generally low in nutrient content. Vitamin supplementation in alcoholic beverages can play a role in mitigation of these deficiencies, e.g., some Danish beer brands contain vitamins (B6 in addition to thiamine) to ‘normalize’ blood thiamine levels in those with alcoholic neuropathy [64]. However, individuals with alcoholism require levels of thiamine supplementation much higher than that required from the average diet, so control of dietary intake alone is not the whole story [61]. In addition to thiamine deficiency AUD is associated with other malnutrition and other micronutrient deficiencies which are comprehensively reviewed by McLean et al. [65].

Alcohol use can also cause thiamine deficiency by disrupting absorption in the gastrointestinal tract. Alcohol damages the mucosa of the gut and reduces intestinal thiamine transport. Studies in both humans and rodents have demonstrated that thiamine is transported via an active sodium independent transporter and therefore requires both energy and a normal pH level [66,67,68], both of which are reduced in alcoholism. Additionally, thiamine absorption can further be depleted by diarrhoea or vomiting which are common occurrences in alcoholism. It is also important to note that thiamine absorption in the gut can be altered by several genetic variants that affect thiamine transport and metabolism [69].

Alcohol also hinders the ability of cells to utilize the thiamine. Thiamine requires phosphorylation by thiamine pyrophosphokinase to be converted to its active co-enzyme form. Thiamine pyrophosphokinase is inhibited by alcohol, which also increases the rate of thiamine metabolism [63]. This phosphorylation step requires magnesium as a cofactor, which is also depleted in alcoholism [70]. Cumulatively, alcoholism leads to thiamine deficiency via the reduction of intake, uptake, and utilization.

Chronic thiamine deficiency ultimately leads to neurotoxicity. As previously mentioned, thiamine is an essential cofactor required for the synthesis and function of several essential enzymes. One of these enzymes is transketolase which is required for glucose breakdown via the pentose phosphate pathway. This pathway produces several essential products. The first is Ribose-5-Phosphate which is required for the synthesis of nucleic acids and other complex sugars. The second is nicotinamide adenine dinucleotide phosphate (NADPH) which is required in the assembly of coenzymes, steroids, fatty acids, amino acids, neurotransmitters, and glutathione [61]. The reduction in production of these factors in addition to thiamine deficiency interrupts the cells’ defense mechanisms, notably the ability to reduce reactive oxygen species (ROS), leading to cellular damage. This damage then triggers apoptosis (cell death). Another mechanism by which thiamine deficiency leads to cytotoxicity is by affecting carbohydrate metabolism leading to the reduction of the enzyme α-Ketoglutarate Dehydrogenase, leading to mitochondrial damage, which in turn induces necrosis [61].

The brain is the most energy-utilizing organ in the body, necessitating a constant supply of energy to function. Without the breakdown of glucose and the subsequent production of essential molecules, thiamine deficiency leads to brain dysfunction and degeneration. The most extreme outcome of alcohol-induced thiamine deficiency-related neurotoxicity is the development of Wernicke–Korsakoff Syndrome (WKS). WKS refers to the closely associated conditions of Wernicke’s encephalopathy (WE) and Korsakoff’s Psychosis (KP). WE is an acute, but reversable condition characterized by confusion, oculomotor disturbances and ataxia [61]. However, these are all rarely seen together so a high index of clinical suspicion should be maintained. WE is caused by thiamine deficiency induced energy deficits and glutamate increases leading to cytotoxicity [63]. WE can develop into non-reversable brain damage (KP) relating to behavior abnormalities and memory impairments.

Both WE and KP are well characterized disorders associated with a distinct clinical and neuropathological presentation. More broadly neurological structural and functional consequences of alcoholism are called alcohol-related brain damage (ARBD). Reduced cognitive functioning is prevalent in between 50% and 80% of individuals with AD [71]. These deficits are often transient [72] and are not normally as severe as those observed in patients with WE or KP [73], but instead it has been hypothesized that ARBD may evolve to KP via progressive brain damage and associated cognitive impairment [74]. Despite individual variations in severity, it is well established that thiamine deficiency leads to neurotoxicity with negative consequences for cognitive functioning.

### 2.2. Neurotoxicity of Acetaldehyde

Alcohol is metabolized to acetaldehyde, via the action of alcohol dehydrogenase (ADH), CYP2E1 and catalase. Acetaldehyde is known to be toxic active metabolite, it is implicated in; the induction of alcoholic cardiomyopathy [75], the development of cancers [76] and to have some neurobehavioral effects [77]. During intoxication the production of acetaldehyde can cause flushing, increased heart rate, dry mouth, nausea and headache [78]. Notably, Acetaldehyde contributes to toxic effects of chronic alcohol on the brain leading to neuronal degeneration [79]. Acetaldehyde induces cell damage and cytotoxicity by inducing DNA malfunction and protein adducts [78]. Additionally, this protein adduct formation can also induce an immune response which can further damage tissues.

### 2.3. Alcohol and Neuroinflammation

In addition to thiamine-deficiency and acetaldehyde related toxicity, alcohol can also cause damage via peripheral and neuro-inflammatory mechanisms. Studies in rodents have demonstrated that alcohol stimulates intestinal inflammation by irritating the stomach and gut, causing the release of the nuclear protein high-mobility group box 1 (HMGB1), which subsequently activate Toll-like receptor 4 (TLR4) and makes the gut “leaky” [80]. This makes alcohol and endotoxins more likely to cross the lining of the gut and travel via the circulation to the liver. Further alcohol metabolism and increases in bacteria cause the liver to produce inflammatory factors such as pro-inflammatory cytokines [81]. This cumulatively increases levels of circulating pro-inflammatory cytokines which can cross the blood brain barrier (BBB) and cause inflammation in the brain [82].

Alcohol also induces neuroinflammation via alterations in neurotransmitter levels. Alcohol is known to increase glutamate levels via the inhibition of the N-methyl-D-aspartate (NMDA) receptor [83] and its cellular action on glutamatergic neurons [84]. Elevated levels of glutamate in a rodent binge drinking model are associated with increased microglial activation in the hippocampus [85]. In addition, alcohol also activates the body’s main stress response system, the hypothalamic–pituitary–adrenal (HPA) axis. When activated, the HPA axis results in the release of corticotropin-releasing hormone (CRH), which acts to suppress peripheral inflammation but increases neuroinflammation via a complex regulation of NK-cells, [81] and by potentiating NF-κB activation in the rodent prefrontal cortex [86]. This combination of increased glutamate and CRH levels enhance the ability of alcohol to induce neuroinflammation and cause subsequent tissue damage.

Alcohol can also directly induce neuroinflammation via the activation of resident neuro-immune cells (microglia and astrocytes). Microglia respond to pathogens, tissue damage, cell death and degeneration. They can respond in a pro- or anti- inflammatory way and depending on the type of activation will produce neurotoxic or neuroprotective mediators [81]. Once activated, alterations in microglial gene expression can potentially lead to increases in inflammatory mediators such as cytokines, glutamate and reactive oxygen species (ROS). These mediators are associated with microglial-dependent neuronal loss [87]. Microglial activation is characterized not only by changes in gene expression, but also by characteristic changes in morphology. Microglia normally survey the brain tissue in a ramified shape with several projecting processes, however once activated the processes shrink and thicken, and the cells gradually become ameboid in shape [88]. Chronic alcohol consumption can therefore cause the de-regulation of microglial activation. This in turn can lead to degeneration of brain tissue and is likely associated with brain volume loss [46], as covered in Section 1.

Alcohol is thought to activate microglia partially via TLR4 receptors, indeed TLR4 deficiency protected against alcohol induced glial activation and neurotoxicity in a rodent model of chronic alcohol consumption [89]. Several studies have investigated the effect of alcohol administration on microglia. Analysis of post-mortem brains of patients with Alcohol Use Disorder showed in increase in microglial markers (Iba1 and GluT5) compared with controls [82]. Binge alcohol administration in adolescent rats established microglial proliferation and morphological changes [90]. However, the activation was described as only partial due to the lack of alteration alcohol had on levels of MHC-II or TNF-α expression. Conversely, microglial activation and neurodegeneration were clearly shown in rats exposed to intermittent alcohol treatment [91]. Indeed two-photon microscopy has been used to demonstrate the rapid response of microglia to even single acute alcohol exposure [92]. Microglial activation has also been investigated in response to heavy session intermittent drinking in rodents [93]. It has been suggested that peripheral inflammation could be caused by stimulation of systemic monocytes and macrophages or by causing gastrointestinal mucosal injury [93]. This innate response was linked to the perpetuation of the immune cascade via microglial activation which produces neuroinflammation [94] this, in turn has been shown to affect cognitive function [93]. Initial transcriptome studies indicated that alcohol increased levels of TSPO (18 kDa translocator protein, that is upregulated in activated microglia). These findings were supported by PET studies performed in baboons [95]. However, when TSPO binding was analyzed using PET in alcohol dependent individuals and individuals undergoing detoxification these findings were not replicated [96,97]. Cumulatively, this evidence suggests that alcohol is clearly an activator of microglia, and as previously described upregulation of microglial activation can result in neurotoxicity. However, the extent of alcohol induced microglial activation may well be dependent on the extent and pattern of alcohol exposure.

### 2.4. Conclusion

In summary, alcohol can contribute to neurotoxicity via thiamine deficiency, metabolite toxicity and neuroinflammation. Alcohol reduces the uptake and metabolism of thiamine, the essential co-factor without which glucose breakdown and the production of essential molecules cannot occur. This leads to neurotoxicity and can lead to the development of conditions of WE and KP. The metabolism of alcohol itself can also lead directly to neurotoxicity as the metabolite acetaldehyde is toxic and can lead to neurodegeneration. Finally, alcohol can lead to neurotoxicity via the induction of both the central and peripheral immune system, causing damaging levels of inflammation.

## 3. Functional Brain Changes

In addition to structural alterations, evidence suggests that chronic exposure to alcohol can lead to functional dysregulation of key brain systems that control behaviour such as reward processing, impulse control and emotional regulation. This likely contributes to the pathophysiology of alcohol misuse and addiction. In recent years, functional magnetic resonance imaging (fMRI) has been used to probe these pathways via blood oxygen level dependent (BOLD) signal in the brain both at rest and during the performance of neurocognitive tasks in an MRI scanner.

### 3.1. Reward Processing

The reward system is in part controlled by the dopaminergic mesolimbic pathway. Originating in the ventral tegmental area. (VTA), dopaminergic projections extend through the striatum and prefrontal regions of the brain. The reward system is responsible for goal-directed behavior by means of reinforcement and responds to conventional rewards such as food and money, as well as all known drugs of abuse. Drugs of abuse, including alcohol, interact with and influence this system and several fMRI paradigms have been developed to probe such effects. One of the most commonly used to probe non-drug related reward sensitivity is the monetary incentive delay (MID) task [98], whereas to measure drug-related reward, cue-reactivity tasks are usually employed [99]. Most commonly these tasks consist of presenting the individual with static or video imagery of a ‘cue’, typically drug or related paraphernalia, however, smell and taste can also be used.

In response to alcohol-related cues, abstinent alcohol-dependent individuals demonstrate an increased BOLD signal in reward-related fronto-striatal brain regions compared with healthy controls, notably prefrontal cortex, ventral striatum (VS), orbitofrontal cortex (OFC) and anterior cingulate cortex (ACC), which is often associated with increased craving [99]. In contrast, a blunted BOLD response to anticipation of non-drug rewards has been observed in the VS and dorsal striatum (DS) [100]. Together, these findings suggest that in alcohol dependence the reward system attributes excess salience to alcohol-related stimuli while simultaneously responding less to conventional rewards.

Interestingly, evidence suggests that dysregulation of the reward system in abstinent alcohol-dependent individuals can be ameliorated by pharmacological intervention. For example, naltrexone, a µ-opioid receptor antagonist, can attenuate the increased BOLD response to alcohol-related cues in the putamen and reduce risk of relapse [101].

Alcohol-related functional differences in the brain are not exclusively observed in dependent individuals. When comparing the neural response of light (consuming ~0.4 drinks per day) and heavy (consuming ~5 drinks per day) drinkers to alcohol cues, light drinkers have been found to have a higher BOLD signal in VS, while heavy drinkers show an increased BOLD signal in DS [102]. The DS response in the heavy drinkers suggests the initiation of a shift from experimental to compulsive alcohol use during which a shift in neural processing is thought to occur from VS to DS control [103]. However, such cross-sectional studies are unable to establish whether such differences are prodromal or consequential of alcohol exposure. A recent longitudinal study in adolescents showed that blunted BOLD response to non-drug reward was predictive of subsequent problematic alcohol use [104]. Similarly, college students who transitioned from moderate (consuming less than 30 drinks per month) to heavy (consuming more than 30 drinks per month) alcohol consumption exhibited hyperactivity in the striatum, OFC, ACC and insula in response to alcohol-related cues compared with those whose alcohol consumption did not alter over time [105]. These results suggests that certain functional differences in reward processing may predate problematic alcohol consumption.

### 3.2. Impulsivity

Impulsivity, a term used to describe a lack of inhibitory control characterized by reckless behavior in the absence of premeditation, has multiple domains including choice, trait, and response inhibition [106]. Increased impulsivity is thought to be a determinant and a consequence of alcohol use [107]. At the behavioral level, alcohol intoxication has been shown to increase risky behaviors such as risky driving, criminal behavior, and sexual promiscuity [108], whilst trait impulsivity has often been found to be increased in alcohol dependent individuals [109].

To probe impulsiveness through fMRI, response inhibition tasks are commonly used, such as the Go/no-go (GNG) task and Stop Signal Task (SST). Several longitudinal studies have probed response inhibition in adolescent drinkers. Such studies have found that adolescents who later transitioned into heavy drinking had lower BOLD activation at baseline and increased activation in frontal regions when subsequently drinking heavily compared with continuous non-drinkers [110,111]. This supports the role of impaired response inhibition as a risk factor rather than a consequence of alcohol consumption.

Choice impulsivity, the tendency to make choices that lead to suboptimal, immediate or risky outcomes is often measured using a delay discounting task to assess an individual’s preference for a smaller, immediate reward compared with a larger, delayed reward [112]. The literature regarding the effects of alcohol on choice impulsivity is varied with findings that alcohol (0.7 g of alcohol/kg body weight) consumption decreased choice impulsivity in non-dependent drinkers [113], whereas another found alcohol (0.2–0.8 g of alcohol/kg body weight) intoxication increased choice impulsivity [114]. Individuals who scored higher in trait impulsivity measures exhibited greater choice impulsivity than their lower trait impulsive counterparts [115].

### 3.3. Emotional Regulation

Altered emotional processing has been found both during alcohol intoxication and dependence and appears to worsen as consumption increases. At the behavioral level, binge drinkers, as defined by scoring in the top third of the Alcohol Use Questionnaire (AUQ), report reduced positive mood and alcohol dependent individuals are more likely to interpret disgusted faces as angry faces and demonstrate a bias for fear recognition in facial expressions when fearful faces are morphed with happy, surprised, sad, disgusted or angry faces [116].

The brain mechanisms of emotional regulation can be measured using imagery tasks where participants are shown either faces expressing emotions or evocative/aversive images designed to evoke emotional responses. In heavy drinkers, who consume more than 15 drinks a week, a blunted BOLD response to fearful faces in the amygdala, a region of the brain involved in fear conditioning and stress responses, has been found to be associated with drinking level in the previous 3 months and higher scores on an obsessive-compulsive drinking scale [117]. These findings suggest that acute intoxication diminishes one’s ability to process emotional information accurately and that this may be perpetuated with heavy alcohol consumption.

### 3.4. Resting State Functional Connectivity

Resting state functional connectivity (RSFC) is a technique that quantifies connections between brain regions based on temporal correlation of BOLD signal change. In a recent UK BioBank study of 25,378 individuals, increased within-network connectivity was identified within the default mode network (DMN) in those with higher alcohol consumption [46]. The DMN is believed to be involved in the processing of self-awareness, negative emotions, and rumination, so increased connectivity within this network may infer a decreased responsiveness to external incentives and increased rumination towards alcohol-related cues [118].

Interestingly, in abstinent alcohol-dependent individuals, RSFC was increased between the amygdala and the substantia nigra/VTA and associated with increased lifetime exposure to alcohol [119]. Such differences in abstinent individuals can suggest a pathological change in brain function after chronic exposure to alcohol or a mechanism for successful abstinence. The latter proposal is corroborated by Beck at al., 2012 [120] who found that hyperconnectivity between these regions during a cue reactivity task was associated with successful maintenance of abstinence.

### 3.5. Conclusion

fMRI studies have allowed us to identify the effects of alcohol use and dependence on brain function as well as vulnerability to heavy use. Typically, exposure to alcohol sensitizes the reward system to alcohol related cues, interferes with the processing of non-drug reward, increases impulsivity, and disrupts emotional regulation. However, the findings discussed here also highlight the variability of individual differences in the presence and magnitude of such neurocognitive deficits which may be driven by exposure, trait factors or abstinence. Finally, an important caveat to much of the present evidence is the generalizability of small cohort cross-sectional studies. To better characterize brain function and behavior following exposure to alcohol both acute and chronic, as well as improve treatment outcome and reduce risk of relapse, it is imperative that large-scale studies with longitudinal designs are conducted. This information is critical for development of alcohol regulation and abuse prevention.

## 4. Neurochemical Dysfunction in Alcoholism

Neuroimaging studies have also dramatically advanced our understanding of the brain’s response to alcohol and the neurochemical basis of alcohol dependence. Positron emission tomography (PET) and single photon emission computed tomography (SPECT) use radiotracers that bind specifically to key receptors of interest, to quantify receptor location and availability. Neurotransmitter release can also be indirectly quantified using PET, through measurement of the amount of tracer that is ‘displaced’ from the receptor when endogenous neurotransmitter is released in response to a pharmacological (or other) challenge. Such techniques have been instrumental in the investigation of key neurotransmitter systems and identification of molecular dysfunction in the human brain. The use of PET to study the effects of chronic alcohol consumption has advanced our understanding of reward mechanisms, neuroadaptations resulting from chronic use that led to tolerance and withdrawal and has identified key regions and circuits implicated in loss of control and motivation to drink. This section summarizes PET studies that investigate the key neurotransmitter systems and review the evidence in case-control studies (summarized in Table 1).

### 4.1. Dopamine

The dopamine system has been the most extensively studied neurotransmitter system in addiction and several targets in both pre- and post-synaptic locations have been evaluated for their respective roles in alcoholism. Dopamine receptor number (availability) and dopamine release can both be measured, in receptor availability and neurotransmitter challenge PET studies, respectively. In studies investigating receptor availability, the key target to date has been the dopamine D2 receptor in the striatum, primarily using the antagonist radiotracer [^11^C]Raclopride which binds selectively to both D2 and D3 dopamine receptors (hereafter referred to as DRD2/3). Data from 7 studies with 105 alcohol dependent individuals and 113 healthy controls were compared in a meta-analysis, revealing an overall reduction in DRD2/3 availability in the alcohol dependent group with an effect size of −0.78 (95% CI, −1.21, −0.35, *p* < 0.001) [121]. Lower DRD2/3 receptors in alcoholism in turn has been associated with decreased metabolic activity in prefrontal brain regions necessary for cognitive control and executive functioning, as assessed with the radiotracer [^18^F]Fluorodeoxyglucose (FDG) [122]. This could explain the vulnerability of such individuals to both compulsive and impulsive drinking, due to disrupted self-regulation [123]. Combined PET/fMRI studies have indicated that reduced striatal DRD2/3 availability was associated with greater frontal BOLD reactivity to alcohol-induced cues [124] indicating a relationship with reward processing. Moreover, the severity of clinical impairment has been shown to correlate with cortical hypometabolism as measured with FDG PET in alcoholism [125], providing several potential functional implications for D2 and/or D3 receptor loss.

Using other dopaminergic tracers, reduced levels of DRD2/3 availability and dopamine synthesis capacity, as measured by [^18^F]DMFP and [^18^F]DOPA, respectively, have been showed to be associated with increased craving and relapse [126], suggesting these receptors have prognostic value and may represent a target for drug development through upregulation of dopamine receptor function or dopaminergic transmission. To support such hypotheses, Rominger et al. identified that DRD2/3 receptor numbers, as assessed with [^18^F]Fallypride, recovered by 30% in individuals who successfully abstained from alcohol at one year, to a level comparable with healthy controls [127], whereas in those that relapsed the DRD2/3 receptor levels did not change. [^18^F]Fallypride has additional utility as it can quantify extra-striatal DRD2/3 receptors due to its very high affinity. Accordingly, studies have found lower DRD2/3 availability amongst alcohol dependent individuals in brain regions outside the striatum, such as the thalamus, insular cortex, hippocampus, and temporal cortex in comparison to matched healthy controls [123], although two other studies found no such difference in temporal [128] or frontal binding [129] using this tracer.

The development of novel radiotracers with greater specificity for the dopamine D3 receptor allowed characterization of this subtype which has been shown in preclinical models to regulate alcohol consumption. Notably, no difference in binding in the ventral striatum or caudate or putamen was found, however, there was a significantly higher D3 receptor availability in the hypothalamus that was linked to higher lifetime use of alcohol [130]. Preclinical imaging has identified D3 receptor antagonism as a plausible therapeutic target to ameliorate alcoholism and its potential efficacy as an intervention is currently under investigation using fMRI [131] and combined PET/MR techniques [132].

PET studies using dopamine-sensitive tracers such as [^11^C]Raclopride have successfully been employed to detect changes in dopamine release, demonstrating that dopaminergic deficits exist in alcoholism. For example, amphetamine-induced striatal dopamine release was found to be blunted in alcohol dependence relative to controls [133], highlighting a lower release potential which may explain the reward-deficiency phenomena associated with addiction described earlier.

Dopaminergic function following chronic alcohol consumption has been extensively investigated with several targets for potential therapeutics being discovered. Whilst promising early clinical work has identified some novel pharmacological targets that could be used to treat alcohol dependent individuals, further large-scale studies are required to validate their use and further exploration of dopaminergic dysregulation is warranted to better characterize the extent of pathology induced by alcoholism.

### 4.2. GABA

GABA is the brain’s main inhibitory neurotransmitter and alcohol acutely enhances GABAergic neurotransmission [134]. A host of in-vivo PET imaging studies have observed an association between alcoholism and lower GABA-A receptors in the cortex (medial prefrontal, OFC, parietal, temporal, and ACC) and the cerebellum [135,136,137,138]. These studies have found lower radiotracer binding of between 6–20% using non-subtype selective GABA-A receptor tracers [^11^C]Flumazenil PET and [^123^I]Iomazenil SPECT imaging in alcohol dependence relative to controls. More recently, the alpha-5 subunit selective PET tracer [^11^C]Ro15-4513 identified lower availability of this subunit in the nucleus accumbens (NAc) and hippocampus in abstinent alcohol dependent individuals when compared with matched controls [138]. A functional [^18^F]FDG PET study investigating the differential effects of a benzodiazepine challenge on cerebellar metabolism indicating that dysregulation of GABA-A receptor may serve as a predisposing trait to alcoholism rather than as a result of chronic alcohol consumption: cerebellar hypo-metabolism was evident in those with a positive family history of alcohol dependence compared with family negative history individuals [139].

### 4.3. Opioids

The opioid system is acutely involved in the reinforcing effects of alcohol. The µ-opioid receptor (MOR) binds β-endorphins and enkephalins which, in turn, increase dopamine release in the NAc [140]. [^11^C]Carfentanil is a PET tracer that can be used to define MOR receptor availability and is also sensitive to endogenous endorphin release. Endorphin release in the NAc and OFC was measured in light versus heavy drinkers through displacement of [^11^C]Carfentanil following acute alcohol consumption of an alcoholic drink. Changes in OFC binding correlated significantly with problematic drinking and subjective high in heavy drinkers but not in controls [141]. In abstinent alcohol dependent individuals a greater MOR availability in the ventral striatum, as measured by [^11^C]Carfentanil, compared with healthy controls was correlated with a greater craving for alcohol [142]. Increased MOR binding could be due to higher receptor levels or reduced release of endogenous endorphins. It was later postulated that greater [^11^C]Carfentanil binding could be related to reduced β-endorphins in alcoholism. Post-mortem studies have noted a 23–51% reduction in MOR binding [143] in alcohol dependent individuals when compared with controls. Reduced MOR binding in post-mortem tissue could be interpreted as a neuroadaptive response to alcohol-induced release of endogenous β-endorphins in patients with severe alcohol dependence and could explain why naltrexone remains relatively ineffective in this subpopulation [140]. Preclinical data suggests that nalmefene counters alcohol-induced dysregulations of the MOR/endorphin and the KOR/dynorphin system [141]. Drugs that antagonize these receptors, including the licensed drug naltrexone have been found to attenuate alcohol seeking in rats and have been shown to clinically reduce alcohol consumption [144].

### 4.4. Other Neurochemical Systems

The endocannabinoid system is implicated in modulating alcohol rewards [145]. Although limited in scope, one small PET study using [^18^F]FMPEP-d2 reported increased cannabinoid CB1 receptor in alcohol dependence in early withdrawal [146]. A more long-term PET study found that alcohol dependence is associated with widespread reduction of cannabinoid CB1 receptor binding in the human brain and this reduction persists at least 2–4 weeks into abstinence. The correlation of reduced binding with years of alcohol abuse suggests an involvement of CB1 receptors in alcohol dependence in humans [147].

PET studies investigating the serotonin system in alcohol dependence are very limited in number, and so a consensus opinion on their importance has not been reached. Studies have focused on the serotonin transporter (SERT) using [^11^C] DASB, revealing mixed results with some [148,149] reporting increased levels of SERT whereas others have found no difference or reduced levels of SERT [150].

Only recently have radiotracers specific for characterizing excitatory glutamate receptors been developed. Early findings indicate impaired mGluR5 signaling to be involved in compulsive alcohol consumption [151]. These effects are found to be reversible following 28 days of abstinence and so can be viewed as a target to aid withdrawal [152].

### 4.5. Conclusion

The dopamine, GABA and opioid systems are by far the most researched using PET and SPECT imaging techniques to measure neurochemical dysfunction in alcohol dependence, due to the availability of selective radiolabeled tracers for the targets of DRD2/3, GABA-A and MOR receptors, respectively. Well validated tracers for other targets such as those in the serotonergic system do exist, but their use in alcohol dependent individuals is not well characterized. Studies using novel radioligands to assess other receptor targets and neurochemical systems including the endocannabinoid and glutamatergic systems is less advanced, but a few selective tracers do exist. It must be acknowledged that PET/SPECT is somewhat limited as a technique because of its radioactivity meaning that young people and repeat scanning cannot be carried out. Nevertheless, PET/SPECT imaging is still the only way to directly image neurotransmitter receptors and neurotransmitter release (when sensitive tracers are available) in the living human brain. Further studies are required to elucidate receptor changes in response to alcohol consumption and dependence across all known neurotransmitter systems.

## 5. Summary

Chronic alcohol consumption is thought to contribute directly to neurotoxicity via thiamine deficiency, metabolite toxicity and neuroinflammation, leading to the development of serious conditions of WE and KP, and the acceleration of neurodegeneration more generally. In addition, neuroimaging of the brain in response to alcohol dependence has found important structural, functional, and neurochemical differences compared with healthy brains which have shone light on possible chronic effects of alcohol consumption, revealed potential vulnerability markers which may be of clinical relevance, identified prognostic biomarkers associated with relapse and recovery and identified possible biomarkers for drug development. The picture is complex with modulation of brain systems by alcohol differing according to the time course of the disorder, the severity and quantity of alcohol used and with an important role for family history in which genetics also plays a role. The sometimes-contradictory findings could also be related to differences in duration of alcohol abstinence and different characteristics of patients being assessed. Another consideration for PET and fMRI imaging studies in alcohol dependence in general is its association with significant cortical grey matter loss, such that in theory, certain reductions observed in receptor availability or changes in BOLD response may in part be explained by changes in brain volume. Further effort is required to determine the interaction between cortical atrophy and the observed functional and/or neurochemical changes.

There is evidence of gender- and sex-related differences in consumption of alcohol as well as its effects on the brain [153]. However, neuroimaging studies on the effects of alcohol use and dependence have either excluded women or shown low female enrolment [154]. Consideration of gender- and sex-related effects has also been limited, in part due to a lack of power [154]. Rates of alcohol dependence have increased drastically in women and many of the harmful health effects are more severe and occur more rapidly in women [155]. This underscores the need to examine sex- and gender-related alterations on brain function and structure in alcohol use; improving our understanding of these effects may enable tailoring of pharmacotherapeutic treatments to improve outcomes.

Another important area for further research is to determine whether alterations in brain structure, brain function and receptor availability in alcohol dependent individuals occur as a direct result of alcohol toxicity or whether they represent vulnerability factors for the onset, development or persistence of problematic or dependent drinking, or whether both contribute to the observed changes. Larger prospective studies and those with a longitudinal design are needed to better understand trait markers that may exist prior to the development of addiction and how they may change across the whole trajectory of the disorder to assess causality, and to stratify and target patients most at risk. Continued efforts to identify suitable biological targets to reduce craving, withdrawal, increase cognition and maintain abstinence for those affected are warranted because despite 30 years of neuroimaging and the huge advances in technologies to understand the brain basis of alcohol use disorder, it remains the case that, to date, only three pharmacotherapies are licensed for alcohol dependence and only 9% of such individuals receive such treatment [156].

## Figures and Tables

**Table 1:** Strength of evidence to show direction of effects on receptor radioligand binding in human PET imaging studies in alcohol dependence.

Receptor system	Striatal D2/3	Midbrain D3	Extrastriatal D2/3	GABA-A	µ-opioid	CB1	SERT	mGluR5
Strength of evidence	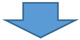	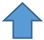	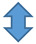	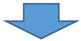	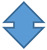	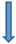	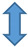	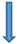

Thickness of arrow indicates the relative strength of evidence of research in the receptor system as assessed by the author based on studies reported in the chapter.
